# FGF21-mediated autophagy: Remodeling the homeostasis in response to stress in liver diseases

**DOI:** 10.1016/j.gendis.2023.05.019

**Published:** 2023-07-13

**Authors:** Wei Shen, Modan Yang, Hao Chen, Chiyu He, Huigang Li, Xinyu Yang, Jianyong Zhuo, Zuyuan Lin, Zhihang Hu, Di Lu, Xiao Xu

**Affiliations:** a Zhejiang University School of Medicine, Hangzhou, Zhejiang 310058, China; b Key Laboratory of Integrated Oncology and Intelligent Medicine of Zhejiang Province, Department of Hepatobiliary and Pancreatic Surgery, Affiliated Hangzhou First People's Hospital, Zhejiang University School of Medicine, Hangzhou, Zhejiang 310006, China; c The Institute for Organ Repair and Regenerative Medicine of Hangzhou, Hangzhou, Zhejiang 310006, China; d Institute of Organ Transplantation, Zhejiang University, Hangzhou, Zhejiang 310003, China; e National Center for Healthcare Quality Management in Liver Transplant, Hangzhou, Zhejiang 310003, China

**Keywords:** Autophagy, Fibroblast growth factor 21, Liver diseases, Stress

## Abstract

Liver diseases are worldwide problems closely associated with various stresses, such as endoplasmic reticulum stress. The exact interplay between stress and liver diseases remains unclear. Autophagy plays an essential role in maintaining homeostasis, and recent studies indicate tight crosstalk between stress and autophagy in liver diseases. Once the balance between damage and autophagy is broken, autophagy can no longer resist injury or maintain homeostasis. In recent years, FGF21 (fibroblast growth factor 21)-induced autophagy has attracted much attention. FGF21 is regarded as a stress hormone and can be up-regulated by an abundance of signaling pathways in response to stress. Also, increased FGF21 activates autophagy by a complicated signaling network in which mTOR plays a pivotal role. This review summarizes the mechanism of FGF21-mediated autophagy and its derived application in the defense of stress in liver diseases and offers a glimpse into its promising prospect in future clinical practice.

## Introduction

Liver is the major organ of nutrient metabolism, which participates in many physiological functions, including energy storage and production, detoxification, immunity, endocrines, *etc*.^1^ Liver disease is characterized by a series of hepatic physiological dysfunction caused by a variety of internal or external pathogenic factors. Numerous factors were reported to trigger liver diseases, such as excessive alcohol consumption, viral infection, drug abuse, and metabolic disorders.^2^, ^3^, ^4^ Therefore, liver diseases can be classified by different etiologies and pathogenesis as acute liver injury, metabolic-associated liver disease (MAFLD), liver cirrhosis, viral hepatitis, and hepatocellular carcinoma (HCC). Both the liver disease itself and the drug or surgical treatment used to treat the liver disease can lead to changes in the homeostasis of the body microenvironment and consequently induce endoplasmic reticulum (ER) stress. The up-regulated FGF21 (fibroblast growth factor 21) can be regarded as a compensatory mechanism in response to ER stress triggered by the unfolded protein response.

FGF21 is one of the 23 members of the FGF superfamily and is recognized as a pivotal regulator in response to stress, such as fasting, high-fat diet, and ischemia.^5^ Lacking a heparin-binding domain, FGF21 can therefore be released from the extracellular matrix and then act as an endocrine hormone on the target tissues via FGF21 receptor 1 (FGFR1).^6^ Beta-klotho is an indispensable single-pass transmembrane coreceptor to strengthen the combination between FGF21 and FGFR1. Beta-klotho is preferentially expressed in the liver, adipose tissues, and nervous system, which may explain the selective metabolic effects of FGF21.^7^ Mounting studies have found the relationships between FGF21 and autophagy. Autophagy is an evolutionary conserved orchestrated program and has attracted more and more attention for its physiological effects on disease progression. There are three types of autophagy: macroautophagy (which is the most studied), microautophagy, and chaperone-mediated autophagy. Facing ER stress, FGF21 is up-regulated by multiple transcriptional factors and then activates various downstream signaling pathways which induce autophagy.

## The interplay between autophagy and liver diseases

### Autophagy is a compensatory mechanism in acute liver injuries

Ischemia-reperfusion injury (IRI) is a noteworthy issue during liver transplantation, partial hepatectomy, and trauma.^8^ In a clinical trial, a frank increase of autophagy in the liver was observed in a subgroup of patients who underwent ischemic preconditioning, while a unique vascular occlusion had almost no effect on autophagy.^9^ Although this study failed to verify the protective effects of ischemic preconditioning, ischemic preconditioning was already demonstrated in many studies to improve the postoperative liver enzyme panel and reduce the postoperative mortality rates.^10^^,^^11^ During liver IRI, autophagy is activated by adverse factors, including increased AMP/ATP ratio, excess production of reactive oxygen species, and calcium overload.^12^ Once the dysfunctional mitochondria, which can produce oxygen free radicals and calcium ions, are too much to be cleared by mitochondrial autophagy, cell death is inevitable.^12^ In a recent study, octreotide-induced protective autophagy in the hepatic IRI mice model was induced by Nrf2-dependent AMPK/PI3K/Akt/mTOR/ULK1 pathways.^13^ However, in another study, hepatic IRI was ameliorated in mice treated with shikonin by suppressing apoptosis and autophagy via activating the PI3K/Akt pathway.^14^ The reason why PI3K acts adversely in regulating autophagy is that class I PI3K inhibits autophagy through PI3K/Akt/mTORC1 pathway, whereas class III PI3K enhances autophagy by inducing Beclin1.^15^ There are also debates about whether autophagy activation initiates or aggravates hepatic IRI since inhibition of mTOR by rapamycin significantly suppressed liver regeneration accompanied by enhanced liver inflammation.^16^

Autophagy activation has also been observed in acute liver injuries caused by different chemicals. As a reaction to reactive oxygen species, ER stress, and mitochondrial damage caused by acute alcohol consumption, autophagy may act as a compensatory mechanism.^17^ When facing acute ethanol exposure, different types of autophagy, such as lipophagy and mitophagy, were reported to be activated.^18^^,^^19^ Both mRNA and protein levels of autophagy-related Atgs were also significantly increased, which is mediated by the transcription factor FoxO3 and hypoxia-inducing factor-1 beta.^20^^,^^21^ The ethanol-induced macroautophagy protects against the toxic effects by selectively removing damaged mitochondria and accumulated lipid droplets.^22^^,^^23^

Similar to alcoholic liver disease, acetaminophen-induced liver injury also leads to liver failure by causing mitochondrial dysfunction and oxidative stress. Various drugs such as chlorpromazine^24^ that activate autophagy were proven to protect against acetaminophen-induced liver injury in mice while other drugs (*e.g.*, chloroquine^25^) that inhibit autophagy increase such liver injury. Mechanistically, autophagy selectively removes damaged mitochondria and acetaminophen-adducts by the PINK1-Parkin signaling pathway.^26^

Autophagy can also ameliorate liver injury by affecting inflammation. Autophagy activated by rapamycin pretreatment was proven to protect against hepatoxicity in acetaminophen-treated mice by inhibiting the activation of NF-kB and NLRP3 inflammasome signaling pathways and the consequent production of IL-1β.^25^ In GalN/LPS-induced acute liver injury, autophagy in macrophage was also significantly enhanced and consequently limited liver injury by reducing the generation of inflammasome-dependent IL-1β.^27^

### Autophagy plays an important role in chronic liver disease

Autophagy deficiency is closely associated with the pathogenesis of a great number of chronic diseases including metabolic syndrome, cardiovascular diseases, MAFLD, *etc*.^28^ MAFLD is the updated nomenclature of NAFLD and was suggested to be diagnosed based on the presence of metabolic dysfunction, which can reflect pathogenesis more accurately and stratify patients more appropriately.^29^^,^^30^ CXC chemokine receptor 3, which indicates autophagosome-lysosome impairment and ER stress, was reported to be significantly up-regulated in both MAFLD mice and patients.^31^

On the other hand, hepatic fat overload caused by insulin resistance and obesity is recognized as the first hit of MAFLD according to the two-hit hypothesis.^32^ An excess of free fatty acids in the cytoplasm is the largest contributor to liver lipid content and would trigger the formation of harmful bioactive lipids and cause lipotoxicity.^33^ Lipid droplet formation is one of the most important ways to store non-esterified free fatty acids as inert triacylglycerides.^34^^,^^35^ Lipophagy is the key role of autophagy in hepatocytes and is vital in balancing the synthesis and degradation of lipid droplets. The breakup of the balance would lead to the process of fat storage.^36^, ^37^, ^38^

Also, autophagy is tightly related to glucose and lipid metabolism, and the imbalance of glucose and lipid homeostasis is an important cause of MAFLD. One of the mechanisms that lead to liver fat production may be the increase in serum levels of methionine and its metabolite S-adenosylmethionine, which are well-known autophagy inactivators.^39^ Consistently, some severe MAFLD patients were detected with decreased expression of glycine N-methyltransferase.^39^ In addition, hyperinsulinemia caused by insulin resistance can in turn suppress autophagy activity and expression of some key autophagy genes.^40^ Once the impaired autophagy fails to compensate for the insulin resistance, the subsequent hyperinsulinemia in turn damages autophagy, forming positive feedback which eventually accelerates the progression of the MAFLD.

Besides, FGF21 also participated in various chronic liver diseases including chronic liver injury,^41^, ^42^, ^43^, ^44^, ^45^, ^46^ alcoholic liver disease,^47^ hepatocellular carcinoma,^48^, ^49^, ^50^ α1-antitrypsin (α1AT)-related liver disease,^51^^,^^52^ Wilson's disease,^53^ and viral hepatitis^54^^,^^55^ and was reported to play different roles depending on disease and cell (Table 1).

**Table 1:** Autophagy in different chronic liver diseases.

Disease	Cells/organs	Mechanism	Factor involved	Reference
**Chronic liver injury**	Macrophage	Autophagy deficiency promoted proinflammatory macrophage polarization and attenuated liver fibrosis	STAT1	41,42
			MAPK	
	Hepatic stellate cells	Autophagy promoted HSC activation and fibrogenesis	TGF-β1	43, 44, 45
	Sinusoidal endothelial cells	Autophagy participated in handling oxidative stress and the selective loss of autophagy aggravates fibrosis	Nrf2	46
**Alcoholic liver disease**	Hepatocytes	Chronic ethanol exposure impaired autophagy and led to lipid accumulation	Rab7	47
**HCC**	HCC-initiating cells	Autophagy deficiency drove tumorigenesis and promotes the development of spontaneous liver tumors	mTOR	48, 49, 50
			p62	
			Nrf2	
**α1AT-related liver disease**	Liver	Mutant ATZ proteins were degraded by autophagy	AMPK	51,52
			ULK1	
**Wilson's disease**	Liver, brain	Autophagy activation prevented copper-induced apoptosis	ATG7	53
			ATG13	
**Viral hepatitis**	Liver	Autophagy was upregulated by hepatotropic viruses and was necessary in virus replication		54,55

Abbreviations: HCC, hepatocellular carcinoma; α1AT, α1-antitrypsin; ATZ, antitrypsin Z protein.

### FGF21 regulation in response to stress

Liver is the pivot of metabolic activities and undertakes vital synthesis and secretory functions in preserving whole-body homeostasis, which requires abundant ER and mitochondria. Thus, ER stress and normal mitochondrial function play important roles in adapting varieties of extracellular and intracellular disturbances. When facing disturbances like hyperlipidemia and inflammation, ER engages the unfolded protein response to reduce the secretory protein load, enhances ER protein folding, and increases clearance capacity.^56^ Crosstalk among the unfolded protein response, autophagy, and mitochondrial function either restores cellular homeostasis or commits to cell death.^57^

Faced with ER stress, the most well-known signaling pathway that up-regulates FGF21 in the liver is PKR-like ER kinase (PERK)-eukaryotic translation factor 2α (eIF2α)-activating transcription factor 4 (ATF4) signaling pathway.^58^, ^59^, ^60^, ^61^ The metabolic effects of autophagy or its deficiency affect the metabolism of distant organs or the whole body through the endocrine effect of FGF21. While systemic ablation of ATG7 in mice, a core gene encoding a protein that is indispensable to classical degradative autophagy, leads to embryonic or perinatal lethality, in mice with liver or skeletal muscle-specific deletion of Atg7, FGF21 expression was increased by autophagy deficiency through induction of activating ATF4.^62^ FGF21 was proven to increase both in the liver of patients suffering from steatosis and mouse models of obesity or NAFLD where ER stress was triggered.^59^ Unfolded protein response is also found to be cross-regulated with integrated stress response. Phosphorylation of elf2α regulates all the stress signaling pathways of integrated stress response.

Under extreme conditions like fasting or starvation, transcription of the hepatic Fgf21 gene is activated by PPARα in response to increases in fatty acids and ketones,^63^^,^^64^ which explains why FGF21 was first dubbed as a starvation hormone. With the deepening of research, more and more FGF21 transcriptional activation sites have been explored in recent years.

Nuclear factor erythroid 2-related factor 2 (NRF2) is an upstream transcription factor of FGF21 which plays an important role in regulating cellular oxidative stress response and protecting against toxic and oxidative stress.^65^^,^^66^ Using CRISPR/CAS9 genome library screening, it is found that by binding to NRF2, FGF21 stabilizes NRF2 and reduces its ubiquitination, generating a positive feedback loop in sorafenib-resistant HCC.^66^ In db/db mice, both plasma FGF21 level and hepatic FGF21 expression were observed to be up-regulated in the administration of CDDO-Im (a potent Nrf2 inducer). The up-regulation of FGF21 was reversed in Nrf2 knockout db/db mice.^67^ Nrf2 also enhanced glucose- and lipid-metabolism-related gene expression in adipose tissues in Keap1-knockdown db/db mice.^67^

*In vitro*, leptin increased FGF21 expression in HepG2 cells, which was mediated by STAT3 activation.^68^ Other signaling pathways that have been discovered to activate FGF21 expression include proliferator-activated receptor gamma coactivator 1-alpha (PGC-1α)- sirtuin1 (SIRT1) axis,^69^ phosphatidylinositol 3-kinase (PI3K)-Akt axis,^70^ *etc*. Interestingly, FGF21 levels increase with age independently of body composition in healthy individuals, probably resulting from the different metabolic demands of the skeleton.^71^ Moreover, retinoic acid receptor beta regulates FGF21 expression.^72^

**Figure 1:**
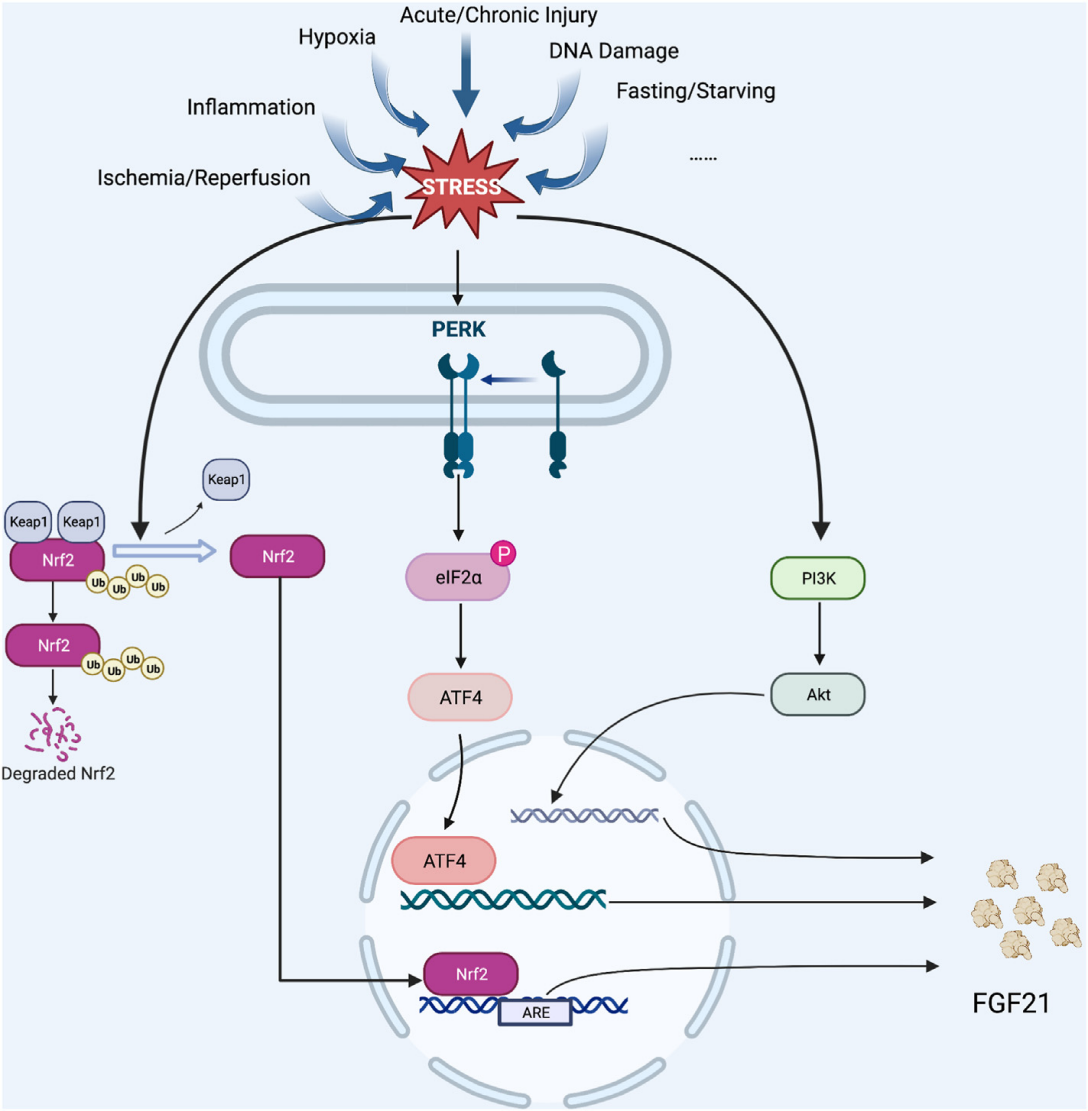
Induction of FGF21 expression by stress-induced activation of eIF2α, Nrf2, and PI3K can be regarded as a compensatory mechanism in response to stress. ARE, anti-oxidant response elements; ATF4, activating transcription factor 4; Keap1, Kelch-1ike ECH- associated protein l; Nrf2, E2-related factor 2; PI3K, phosphatidylinositol3-kinase.

Given the role of FGF21 in maintaining homeostasis and promoting regeneration, which is mostly through the regulation of autophagy, it is likely that FGF21 activation is a compensatory protective effect in response to stress. Figure 1 exhibits some most reported signaling pathways of stress-induced FGF21 expression.

### The mTOR pathway in FGF21-mediated autophagy

The mammalian target of rapamycin (mTOR) is an evolutionarily conserved atypical serine/threonine protein kinase and is one member of phosphatidylinositol kinase-related kinase.^73^ By integrating a variety of extracellular signals such as nutrition, energy, and growth factors, mTOR participates in gene transcription, protein translation, ribosome synthesis, and other biological processes and plays an extremely important role in cell growth,^74^ apoptosis, autophagy^75^ and metabolism.^76^ mTOR complex1 (mTORC1) and mTORC2 are two forms of mTOR according to the distinct companion proteins they bind. Characterized with RAPTOR and PRAS40,^77^ mTORC1 is sensitive to rapamycin and blocks catabolic processes such as autophagy at the post-translational and transcriptional levels.^78^^,^^79^

**Figure 2:**
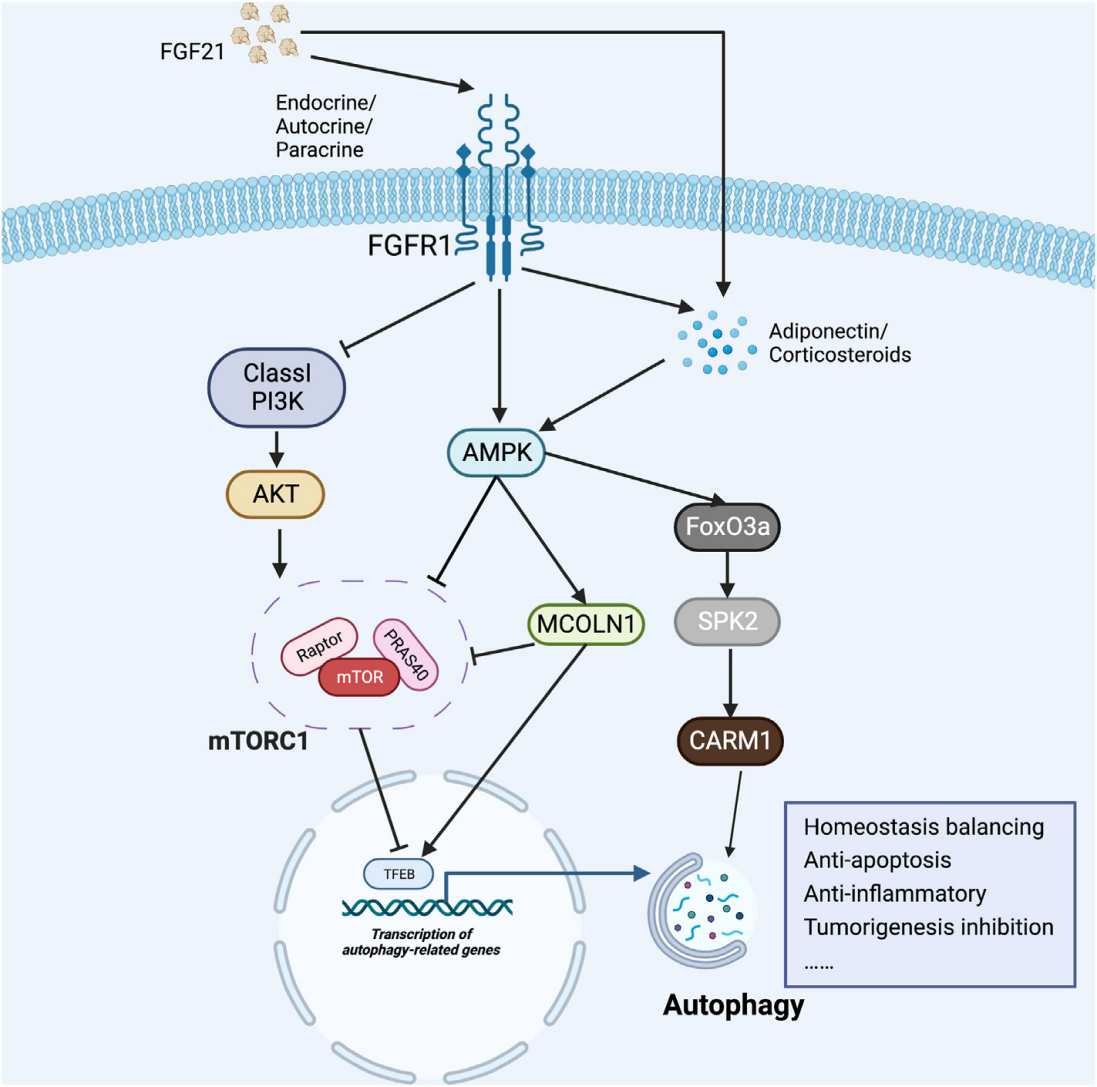
mTORC1 plays a pivotal role in FGF21-mediated autophagy. FGF21 can either activate the AMPK directly through FGFR1/β-klotho signaling or indirectly stimulate the secretion of adiponectin and corticosteroids. Signaling pathways including AMPK, PI3K, and MCOLN can activate the transcription of autophagy-related genes and eventually exert a variety of effects by activating autophagy flux.

As an indispensable cell growth and metabolic regulator, mTORC1 can be regulated by a variety of intracellular and extracellular signals (Fig. 2). AMP-activated protein kinase (AMPK) is a key factor in bioenergy metabolism regulation and can be activated during energy deprivation, hypoxia, and DNA damage by sensing an increase in the AMP/ATP ratio and eventually negatively regulates mTOR.^80^ There is a remarkable overlap between the metabolic responses induced by FGF21 and AMPK activation, such as balancing glucose and lipid homeostasis.^81^ It is reported that FGF21 can not only activate the AMPK directly through FGFR1/β-klotho signaling but also activate the AMPK signaling indirectly by stimulating the secretion of adiponectin and corticosteroids.^81^ In random-pattern skin laps, administration of FGF21 exerted a pivotal effect on flap survival through AMPK-FoxO3a-SPK2-CARM1 and AMPK-mTOR signaling pathways, which enhanced autophagy by mediating the dephosphorylation and nuclear translocation of transcription factor EB (TFEB).^82^ In another study, FGF21 inhibited cell apoptosis and reduced oxidative stress by activating the AMPK-mTOR signaling pathway and therefore increased both the regeneration of the damaged liver and the survival rate of damaged liver cells by augmenting autophagy in zebrafish.^83^ In a recent study, up-regulated FGF21 induced by ischemia/reperfusion could enhance the activity of TFEB in skeletal muscle and consequently protect against oxidative stress since hypoperfusion of limb and tissue edema significantly deteriorated in FGF21-KO mice.^84^ The administration of CC (a specific inhibitor of AMPK) or tacrolimus (a calcineurin inhibitor) obstructed FGF21-mediated activation of autophagy and increased the apoptosis in skeletal muscle, indicating that FGF21 ameliorates IRI through the AMPK-MCOLN1-calcineurin-TFEB signaling pathway.^84^ Although mTOR is not mentioned in this study, it has been reported in another study that MCOLN1 could regulate the local release of Ca^2+^ under nutrient starvation or mTOR inhibition.^85^ However, AMPK can also regulate autophagy in an mTOR-independent way. Increased FGF21 production in muscle induced by exercise intervention can be secreted into the circulation and then enhance the lipophagy by either activating AMPK/ULK1 or inhibiting Akt/mTOR/ULK1 and consequently attenuate the lipid accumulation in the liver.^86^

TFEB is a key transcription factor that regulates lysosomes and is reported by several studies to be activated by FGF21. Although a large proportion of them proved that the activation of TFEB is regulated by mTOR,^82^^,^^87^ reactive oxygen species can also activate TFEB via a lysosomal Ca^2+^-dependent mechanism independent of mTOR.^88^ These suggest that mTOR is an extremely important but not indispensable factor for FGF21-regulated autophagy. FGF21 was also reported to exert protective effects in various diseases such as cardiovascular diseases,^89^ diabetic nephropathy,^90^ and osteoarthritis^87^ through AMPK activation. FGF21 can also up-regulate the secretion of adiponectin, an adipokine that plays a vital role in sensitizing the body to insulin, and hypoadiponectinemia caused by various factors can lead to obesity, type 2 diabetes, and metabolic syndrome.^91^ In adiponectin-treated cells and xenografts, the expression of autophagy-related proteins was significantly up-regulated and effectively inhibited the growth of breast cancer, which is also activated via the AMPK signaling pathway.^92^ P62 is a protein negatively regulated by the adiponectin/AMPK/autophagy pathway. P62 was proven to promote the NF-κB by increasing the phosphorylation of IKKβ through binding to atypical protein kinase C, promoting reactive oxygen species-induced RAS expression, and activating and polyubiquitinating TRAF6.^93^ This indicates the possibility that FGF21 may exert its anti-inflammatory effects also by activating autophagy. However, elevated autophagy flux is not entirely dependent on FGF21 and adiponectin^94^, which needs further investigation.

The AMPK and SIRT1 pathways are both interconnected, and they share several common target molecules.^95^ Sirt1 has long been recognized as a longevity gene that can mediate autophagy in a NAD-dependent way.^96^ Alternate-day fasting was proven to up-regulate FGF21 and further activate the downstream AMPK/SIRT1/PGC-1α pathway, during which mitochondrial biogenesis and autophagy were also stimulated.^97^ In CCl4-induced acute liver injury mice models, exogenous FGF21 treatment effectively protected against hepatoxicity, alleviated inflammation, and up-regulated SIRT1 and autophagy markers such as LC3II and Beclin1. This protective effect could be inhibited by SIRT1 lentit-RNA (which can inhibit SIRT1 expression) both *in vivo* and *in vitro*.^98^ However, in another review concerning Sirtuin's cardioprotective effects, FGF21 was regarded as the downstream rather than the upstream of Sirt1.^69^ These studies suggest that Sirt1 may be involved multi-dimensionally in both FGF21 induction and its subsequent autophagy induction, which need further investigation. Interestingly, Sirt1 was found to also interact with mTOR in recent years, and its effects vary depending on different pathways. Sirt1 overexpression promoted liver inflammation and injury in the cholestatic disease model by activating the mTOR pathway in macrophage, which activated the inflammasome and suppressed autophagy.^99^ *In vitro*, SIRT1/mTOR pathway participated in Pb-induced hepatic lipid accumulation, which could reverse autophagy dysregulation in HepG2 cells.^100^

PI3K is another upstream molecule of mTOR and is also closely related to the Sirt signaling pathway. Sirt1/PI3K/AKT/mTOR pathway is reported to restore autophagy and alleviate hepatocyte senescence in NAFLD mice.^101^ As mentioned above, different subtypes of PI3K act adversely in regulating autophagy. In the FGF21-overexpressing LNCaP cells, increases of Akt, mTOR, and p70S6K phosphorylation levels induced by high glucose were reversed compared with the control group, which confirmed that FGF21 facilitated autophagy by inhibiting PI3K/Akt/mTOR signaling pathway.^102^ The FGF21-mediated autophagy also inhibited prostate cancer tumorigenesis *in vivo*.^102^ However, in another recently-published study, activation of the GATA2/FGF21 axis protected against high-glucose-induced injury through phosphorylating PI3K, AKT, and mTOR in human umbilical vein endothelial cells.^103^

Amino acid is another potent inhibitor of autophagy by activating mTORC1, which is through the PI3K class III (hVps34) pathway or amino acid sensors located in the cytosol or lysosomal membrane.^104^ Pegylated arginine deiminase reversed dyslipidemia, hepatic steatosis, and inflammation in obese mice by promoting mammalian energy expenditure and insulin sensitivity, which depends on the hepatocyte-specific FGF21.^105^ This FGF21-related autophagy-promoting effect was achieved by reducing mTOR phosphorylation.^105^

To sum up, a great number of signaling pathways are involved in regulating FGF21-mediated autophagy, and there are complex crosstalks among them, in which the mTOR pathway plays a pivotal role.

### The promising prospect of FGF21 in clinical practice

**Figure 3:**
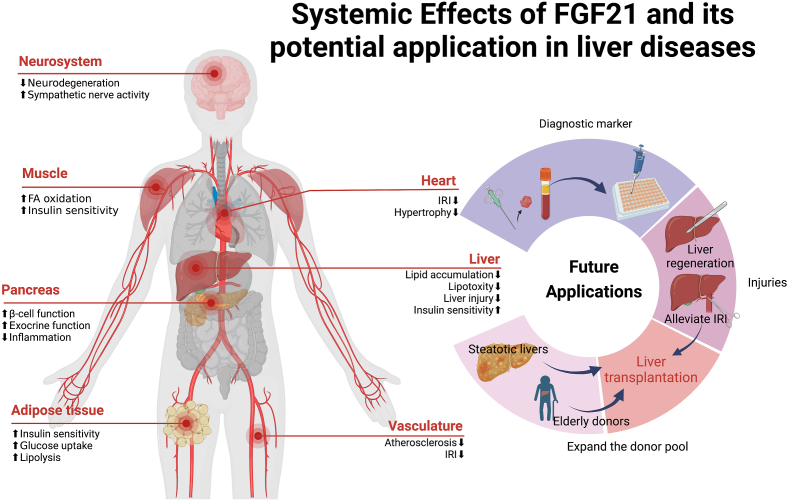
Systemic effects of FGF21 and its potential application in liver diseases. FGF21 plays a protective role in different systems including the liver, pancreas, heart, muscle, neuro-system, adipose tissue, and vasculature. Considering its powerful effects on the liver, FGF21 and its analogues have a promising application in the clinical area including disease diagnosis, liver transplantation, and partial hepatectomy. IRI, ischemia-reperfusion injury; FA, fatty acid.

Given the multiple beneficial effects of FGF21, it is not surprising that FGF21 and its analogues will be substantially used in clinical practice in the near future (Fig. 3). Since FGF21 can be considered a marker of ER stress, it can be used as an efficient diagnostic biomarker for a variety of diseases including alcoholic cardiomyopathy,^106^ muscle dysfunction,^107^ and liver diseases like NAFLD^108^ and HCC.^109^ The increased FGF21 in these pathologies likely reflects hypertriglyceridemia, hyperinsulinemia, and pericardial fat accumulation in the liver.^110^^,^^111^ Ye et al revealed that serum FGF21 was markedly elevated well before the IRI-induced massive hepatocellular damage during liver transplantation. The peak serum levels of FGF21 at 2 h are closely related to the magnitude of the increase in aspartate aminotransferase and aspartate transaminase levels.^112^ Also, in a study among pediatric recipients who underwent liver transplantation, FGF21 levels were higher in patients than in controls, and patients displayed increased whole-body synthesis and decreased intestinal absorption of cholesterol. However, serum cholesterol levels showed no significant difference.^113^ There is also a positive correlation between plasma FGF21 and the severity of steatohepatitis, particularly fibrosis, in patients with non-alcoholic steatohepatitis.^114^ However, in obese mice, a humanized bispecific antibody that can specifically activate the FGF21 receptor complex has lower uptake on a per gram basis than non-obese mice, probably due to the reduced expression of receptors in white adipose resulting from obesity.^115^ This suggests that FGF21 may also have resistant effects similar to insulin resistance in diabetes and therefore may not be suitable for measuring the development and severity of chronic diseases in some situations, which needs to be further investigated.

Given the multiple beneficial effects of FGF21, it is not surprising that FGF21 and its analogues will be substantially used in clinical therapy in the near future. Although several studies have reported FGF21's adverse effects on bone in rodents, administration of pegbelfermin, a PEGylated FGF21 analogue, showed no PGBF-related bone toxicity effects in skeletally-mature monkeys after one-year dosing, which suggests FGF21's safety clinical application in adult humans.^116^

Although lacking direct evidence in attenuating liver IRI, FGF21 was proven to alleviate microvascular damage by TFEB-mediated autophagy after limb IRI.^84^ Similarly, FGF21 secreted by the liver and adipose tissue also exerts a cardiac endocrine protective effect in myocardial ischemia.^117^^,^^118^ Considering IRI is the major cause of liver dysfunction after liver transplantation,^8^ the potential protective effects of FGF21 in liver grafts are worth exploring. In a liver injury model in zebrafish, FGF21 administration significantly inhibited cell apoptosis by up-regulating antiapoptotic genes and down-regulating proapoptotic genes and was also testified at the protein level. FGF21 also efficiently elevated the level of superoxide dismutase, a key enzyme against oxidative stress, which could reduce the activity of aspartate aminotransferase, aspartate transaminase, cysteine aminotransferase, and malondialdehyde and is beneficial to liver regeneration. Not surprisingly, autophagy-related markers such as LC3B were found to be promoted by FGF21 through the AMPK-mTOR signaling pathway. Both the function of anti-oxidative stress and promoting liver regeneration can be blocked using chloroquine, a well-known autophagy inhibitor.^83^ This study indicates FGF21's potential future application after partial liver resection.

In addition, FGF21 variants were engineered to make it more convenient in clinical applications. For instance, Fc-FGF21 (an FGF21 variant fused with Fc) was reported to have a longer circulating half-life and more sustained improvements in multiple metabolic parameters.^119^ In a clinical trial, weekly subcutaneous injection of efruxifermin (a long-acting Fc-FGF21 fusion protein) effectively reduced hepatic fat fraction in non-alcoholic steatohepatitis patients and was generally well tolerated.^120^ LY2405319 is another engineered FGF21 protein that is proven to be effective in a randomized clinical trial in obesity and type 2 diabetes patients, such as reducing body weight, fasting triglycerides, and insulin levels.^121^ LY2405319 could also enhance hepatic mitochondrial function and therefore attenuated non-alcoholic steatohepatitis progression in mice models, which indicates its therapeutic potential in the treatment of advanced liver disease.^122^

## Conclusion

Liver diseases are becoming an increasingly heavy burden worldwide for their high morbidity and mortality. Therefore, finding the pathogenesis and associated treatments of liver diseases is urgent. ER is a key mediator of the acute stress response in the liver and ER stress can consequently induce the expression of the stress-induced hormone FGF21. PERK-eIF2α-ATF4 is one of the most well-known signaling pathways. FGF21 is an endocrine hormone and is reported to regulate glucose and lipid homeostasis, promote liver regeneration, and attenuate ischemia/reperfusion in abundant studies. Recent studies further indicate that those protective effects may be achieved by activating autophagy. Although autophagy can act differently depending on the actual situation, the overall machinery of autophagy tends to protect against injury in the face of ER stress by degrading damaged organelles and recycling ingredients. Hence, pharmacological enhancement of autophagy may exert a potent effect in attenuating clinical symptoms in liver diseases. Considering the multi-dimensional and autophagy-related protective effects of FGF21, it may show more effects in liver diseases, such as attenuating the fatty liver graft injury, protecting against IRI after liver transplantation, and promoting liver regeneration after partial hepatectomy, which can be an attractive research area in the future.

## Author contributions

SW performed the literature review and drafted the manuscript. YMD, CH, HCY, LHG, LZY, YXY, ZJY, and HZH performed the literature review and drafted and revised the manuscript. XX and LD supervised, drafted, and revised the manuscript.

## Conflict of interests

The authors declare that they have no competing interests.

## Funding

This work was supported by the 10.13039/501100012166National Key Research and Development Program of China (No. 2021YFA1100500), the Major Research Plan of the National Natural Science Foundation of China (No. 92159202), the Key Program, National Natural Science Foundation of China (No. 81930016), the Young Program of National Natural Science Funds (China) (No. 82000617), and A Project Supported by Scientific Research Fund of Zhejiang Provincial Education Department (China) (No. Y202250784).

## Data availability

The datasets used and/or analyzed during the current study are available from the corresponding author upon reasonable request.
